# Neutron Diffraction Study of Indole Solvation in Deep Eutectic Systems of Choline Chloride, Malic Acid, and Water

**DOI:** 10.1002/chem.202200566

**Published:** 2022-06-13

**Authors:** Oliver S. Hammond, Ria Atri, Daniel T. Bowron, Karen J. Edler

**Affiliations:** ^1^ Centre for Sustainable Chemical Technologies and Department of Chemistry University of Bath Claverton Down Bath BA2 7AY U.K.; ^2^ Centre for Sustainable Chemical Technologies and Department of Chemical Engineering University of Bath Claverton Down Bath BA2 7AY U.K.; ^3^ ISIS Neutron and Muon Source Science and Technology Facilities Council Rutherford Appleton Laboratory Didcot OX11 0QX U.K.; ^4^ Current address: Department of Materials and Environmental Chemistry Stockholm University Stockholm Sweden

**Keywords:** deep eutectic solvents, green chemistry, heterocycles, neutron diffraction, pi interactions

## Abstract

Deep eutectic systems are currently under intense investigation to replace traditional organic solvents in a range of syntheses. Here, indole in choline chloride‐malic acid deep eutectic solvent (DES) was studied as a function of water content, to identify solute interactions with the DES which affect heterocycle reactivity and selectivity, and as a proxy for biomolecule solvation. Empirical Potential Structure Refinement models of neutron diffraction data showed [Cholinium]^+^ cations associate strongly with the indole π‐system due to electrostatics, whereas malic acid is only weakly associated. Trace water is sequestered into the DES and does not interact strongly with indole. When water is added to the DES, it does not interact with the indole π‐system but is exclusively in‐plane with the heterocyclic rings, forming strong H‐bonds with the ‐NH group, and also weak H‐bonds and thus prominent hydrophobic hydration of the indole aromatic region, which could direct selectivity in reactions.

## Graphical abstract

**The solvation of indole** in choline chloride:malic acid deep eutectic solvents is shown by neutron diffraction with isotopic substitution and atomistic modelling to vary with water content. Latent water does little, whereas adding 2 mole of equivalents of water causes interactions, not with the π‐system of indole, but via hydrophobic hydration and H‐bonding in‐plane with the heterocyclic rings. These results may therefore explain trends in heterocycle reactivity and selectivity.

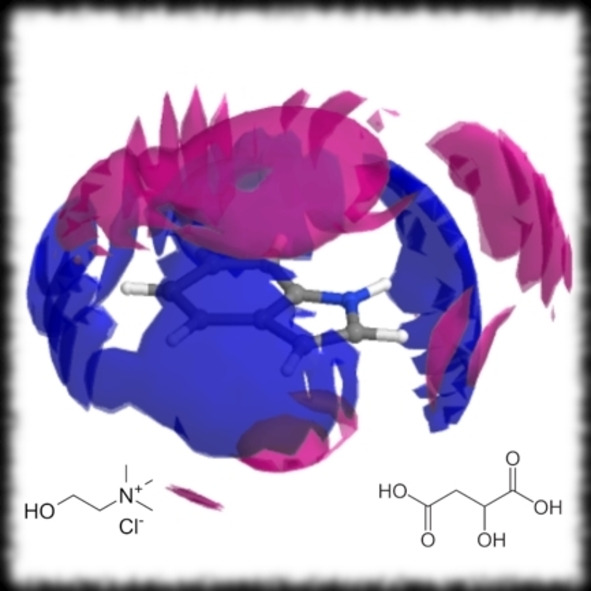

## Introduction

Eutectic mixtures, where melting point depressions are observed for multicomponent systems relative to the pure components, and show a distinctive minimum transition temperature, can safely be categorised as an old and established field of knowledge.^[1]^ In this century however, the field has evolved, following the reconsideration of eutectics as ‘deep eutectic solvents’ (DES).[^2^, ^3^] This seemingly minor redefinition has elicited a cascade of published applications where eutectic mixtures are now applied as the solvent, especially for systems with ionic liquid (IL)‐like character, such as choline chloride‐based mixtures.^[4]^ This is because such systems tend to be very non‐ideal, allowing the liquid phase to be composed of normally solid compounds, at attractively low temperatures.^[5]^ Therefore, entirely new chemistries are likely to be enabled by these eutectic systems and their unique solvation environment.^[6]^ However, the fundamental understanding of the physical chemistry of these systems has lagged behind their deployment, leading to misconceptions regarding their structural state,^[7]^ and overall classification.^[8]^ Pertinently, many reported ‘DES’ are described in idealised ‘magic’ mixing ratios,^[9]^ not at their true eutectic points,^[10]^ which was an issue raised even by Guthrie in the first publication describing eutectics in 1884.^[1]^

There is therefore a clear need to build understanding of the fundamental physical, and especially structural, properties of deep eutectic solvents. This will allow the field of DES to progress beyond iterative and exploratory work, towards rational and informed solvent selection and design, which is typically mooted as one of the major benefits of using DES.^[11]^ Thus, work in the literature has recently targeted DES structure, as was reviewed first by Hammond and Edler,^[12]^ and more recently by Kaur et al.^[13]^ Briefly, the new picture emerging is that the popular choline chloride‐type systems do not have the same repeated domain structure common to ILs,[^7^, ^14^] and are rather more disordered, with each species diffusing at different rates relating to their size and interaction strength,^[15]^ within a loosely‐associated ‘cluster’ in constant flux,[^16^, ^17^] and with a flat potential energy landscape for the various possible interspecies interactions.^[18]^ Initial suggestions from destructive mass spectrometry experiments suggested complex‐ion clustering,^[3]^ and while this may not be the case for the most common DES, there is evidence that it may be the case for more conventionally IL‐like systems such as the ‘Type IV’ metal‐containing DES, which have separated ionic and molecular domains.^[19]^ While most common DES do not form ion complexes or have a nanostructure (a thermodynamically‐stable structural heterogeneity over nanometre length scales), with some careful ‘design’ of the solvent, this nanostructure can be ‘switched on’,^[20]^ and hydrophilic‐hydrophobic domain separation is observed,^[21]^ *à la* ionic liquids.[^22^, ^23^]

It has recently been shown that a hydrophobic solute can be forced to segregate into the nonpolar domain of an amphiphilic DES, on addition of water.^[20]^ In this work we are principally interested in the solvation of a hydrophobic solute in a purely *hydrophilic* DES, and investigating how small quantities of water can affect this. Namely, here we will study the choline chloride‐malic acid ‘natural’ DES system (ChCl : MA).^[24]^ This DES is interesting since both the choline hydrogen bond acceptor (H BA) and malic acid hydrogen bond donor (HBD) component can theoretically be naturally‐sourced. Moreover, malic acid contains both alcohol and carboxylic acid moieties. ChCl : MA has also been studied recently using neutron scattering, making it a good structural benchmark.^[14]^ We will study this solvent in its ‘pure’ state containing only trace absorbed atmospheric H_2_O, and in a ‘hydrated’ state containing 2 mole equivalents of water. The hydrophobe of choice for this study will be the small heterocycle indole. Indole is interesting because it is a hydrotrope with a hydrophobic aromatic region as well as the pyrrole ring H‐bonding site. Indole was first isolated by treatment of *indigo* dye with *ole*um (hence its name), but it is naturally‐occurring as an intercellular signalling regulator in bacteria, and in eukaryotes it is derivatised first into tryptophan and subsequently, serotonin.^[25]^ The reactivity profile of indole is particularly interesting for synthetic chemists, since it is vulnerable to electrophilic aromatic substitution, hydrogenation, cycloaddition and oxidation, while its amine lone pair is unavailable due to aromaticity, whereas the N−H proton is acidic.^[26]^ Indeed, there are numerous studies of reactions of indole‐type compounds in DES,^[27]^ for example, ultrasound synthesis of 3‐substituted indoles,^[28]^ Fischer indolization in DES,^[29]^ and simple^[27]^ and Friedel‐Crafts alkylations.^[30]^ Thus, we consider indole to be an interesting proxy molecule, both for the solvation of metabolites in deep eutectic solvents made from potentially naturally‐sourced components, and for the solvation of small‐molecule organic reaction precursors, in reactions involving indole itself and similar heterocycles.^[31]^ Here, we will therefore use wide Q‐range neutron scattering to measure the static structure factor (S(Q)) for a variety of isotopic contrasts of dilute indole‐in‐DES solutions. The solvation of indole will then be resolved using the Empirical Potential Structure Refinement (EPSR) method, and the models analysed and compared to show the most probable, structure‐defining inter‐ (and intra‐) species interactions.

## Results & Discussion

### Data & fits

**Figure 1:**
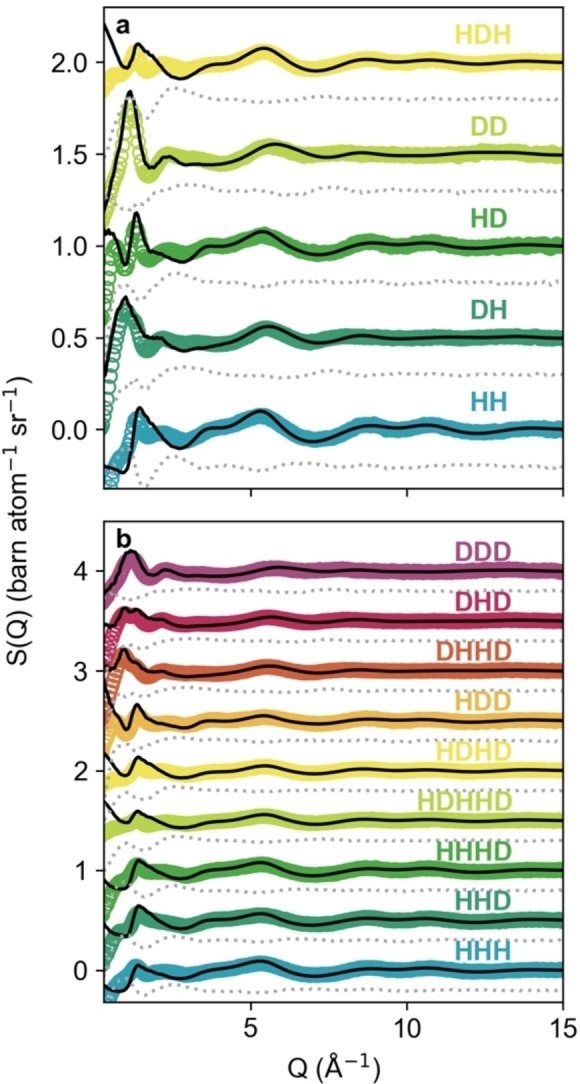
Fits (black lines) & neutron diffraction data (coloured markers) in Q (reciprocal)‐space for the various isotopic contrasts of a) the 0*w* and b) 2*w* systems, with grey dashed lines showing the fit residual.

**Figure 2:**
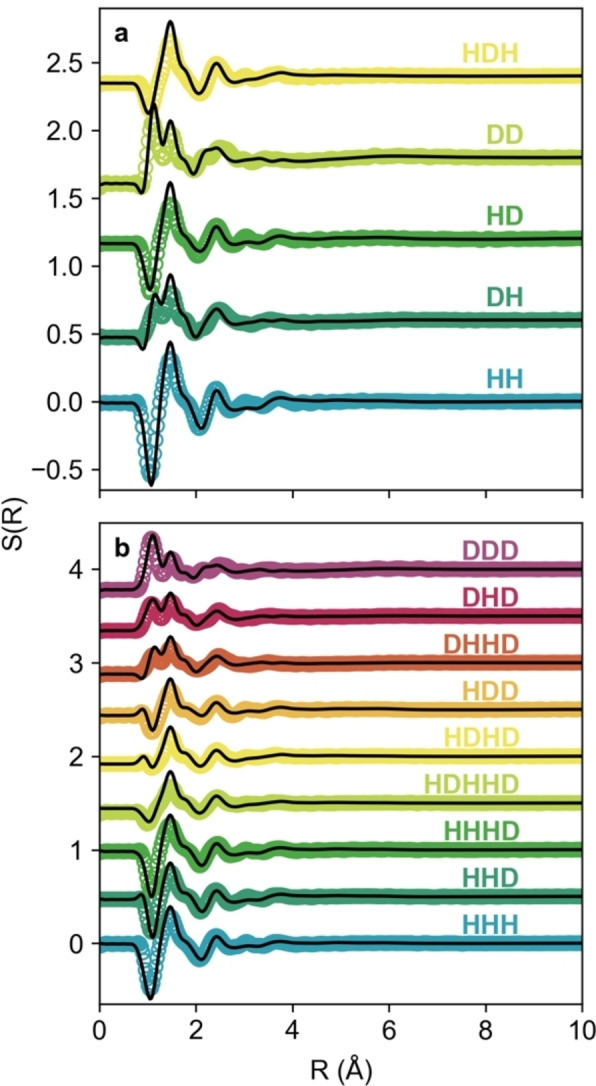
Fits (black lines) and experimental data (coloured markers) in R (real)‐space for the various isotopic contrasts of a) the 0*w* and b) 2*w* systems.

Corrected neutron scattering data and fits, including fit residuals, are shown in Figure 1. In Q‐space, as highlighted by the shown fit residuals (grey dashed lines), there are some minor discrepancies between the fits and data in the low‐Q (<2 Å^−1^) region, but these are mostly due to the difficulties in accurately subtracting the inelastic hydrogen scattering background, and thus this is not considered a significant issue.[^32^, ^33^] Efforts were made to keep samples anhydrous: DES were prepared from choline chloride and malic acid dried under vacuum at 80 °C, mixed immediately before measurement, and kept in desiccators. Despite this, a small quantity of water will always remain, and it was impossible to measure this due to the expensive nature of deuterated samples. Under the same preparation protocol, by analysis of the neutron differential scattering predicted and experimental cross‐sections, samples of ChCl:urea were shown to have a maximum of 0.4 mol % water (ca. 0.1 wt.%).^[17]^ Therefore, a small, measured quantity of water corresponding to 0.1 wt.% was introduced into the ‘pure’ DES models to best reflect that these are ternary systems, since we recognise that *X*_H2O_=0 is challenging to practically achieve.^[34]^ Moreover, even very small quantities of water can markedly change structure (shown here by the chloride‐water RDFs) and the physical properties such as viscosity and, by extension, reactivity and solubility.^[35]^ This low‐level water did not affect the fitting significantly, but the simulation box better reflects the physical reality of the system, and makes the RDF and SDF analysis analogous. Otherwise, despite the presence of the solute, the diffraction data closely match previous reports of the major interaction peak for the malicine DES, at 1.15 Å^−1^.^[14]^ The R‐space transforms (Figure 2) highlight the particularly close match between our model and the measured system, especially for the hydrated malicine‐2*w* system where more contrasts were accessible. From 0–2 Å, the region associated with intramolecular distances and close intermolecular contacts, the fits are near‐perfect. For the region 2–4 Å, fits remain good with only slight discrepancies in intensity, with phasing matching well between fit and data. We are therefore confident that the fits have converged on a structural state closely representing the measured system. Crucially, the diffraction data do not show any low‐Q structure, meaning that there is no systematic phase segregation in these systems, as has been suggested.^[36]^ However, nanostructure and the small‐angle ‘pre‐peak’ can be observed in other, more amphiphilic DES systems.[^12^, ^13^, ^20^, ^21^]

### Bulk structure

**Figure 3:**
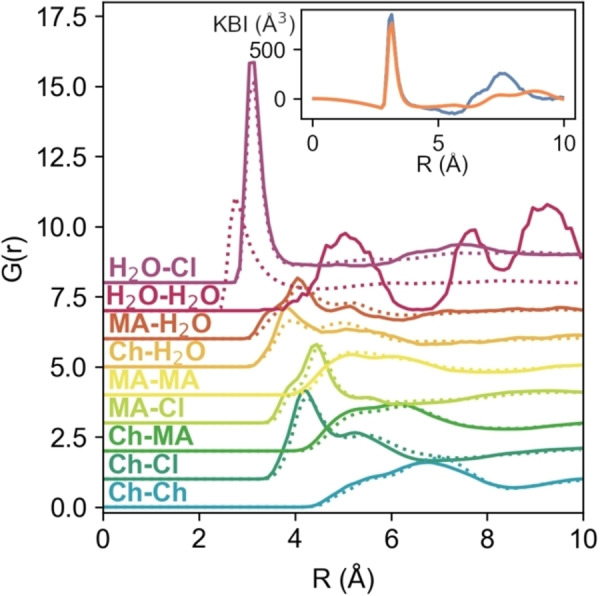
COM‐RDFs of species in the bulk, showing the 0*w* system (solid lines) and the 2*w* system (dotted lines). The H_2_O‐H_2_O RDF for the 0*w* system shows large fluctuations that signify only the poor correlation, due to being effectively at infinite dilution. Inset: The G(r) for the H_2_O−Cl correlation were transformed and used to calculate respective Kirkwood‐Buff Integrals (KBI) of 279 and 56 Å^3^ (per molecule), for the 0*w* (blue line) and 2*w* (orange line) systems, signifying excess hydration of chloride relative to the bulk, especially for the 0*w* system at ∼7.5 Å.

**Figure 4:**
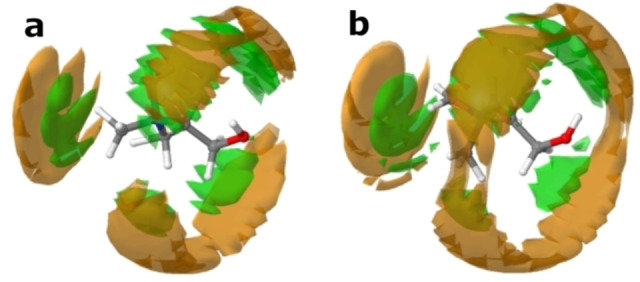
SDFs of a) the 0*w* and b) the 2*w* DES, showing the solvation of choline by malic acid (orange) and chloride (green) isosurfaces, plotted at a surface fraction of the top 7.5 % of configurations.

Initially, we establish that the introduction of the solute, indole, does not greatly perturb the interactions between the DES components themselves. Figure 3 shows the computed centre‐of‐mass radial distribution functions (COM‐RDFs) between the various species present in solution. As is becoming increasingly clear, DES do not seem to form a ‘complex‐ionic liquid’, rather, all species are capable of interacting through electrostatics and both strong and weak H‐bonding. To demonstrate this, and the deviation from ideality, Figure S2 in the Supporting Information shows a comparison between choline solvation in the ideal random non‐interacting case, and the coordination calculated from our EPSR models. The non‐ideality is clear, yet, choline still remains notably associated with chloride and water, and thus the system cannot exist as a complexed IL. Note that, while the water‐water RDF has an unusual appearance for the 0*w* system, this is an artefact of the negligible concentration of water, which is effectively at infinite dilution and thus, with no evidence for clustering, does not experience short‐range direct water‐water interactions. The peaks in the dilute system water‐water g(r) are indicative of more distant correlations, that would arise from the small molecules finding correlated favoured locations within the bulk solvent environment, whereas the associated coordination numbers in Table 1 show that the number of such incidences is extremely low; they are therefore treated as an almost‐zero‐probability flat‐line, which is the result if the simulations are accumulated for much longer. More importantly, the chloride anion is indeed capable of strong interactions with both the ‐OH and ‐COOH groups of malic acid, but the choline‐chloride H‐bond is also present. Each species also interacts with itself, and water, and these systems better fit the description of ‘alphabet soup’, rather than ‘complex ion’.^[18]^ Instead of a complex, the DES components form a ‘cage’ in constant flux, where electrostatics are balanced, and the molecules ‘compete’ to form H‐bonds with one another, with different diffusion coefficients for each.[^15^, ^35^, ^37^, ^38^] This can be visualised, in part, from the calculated SDFs shown in Figure 4. Chloride can associate either with the ‐OH group of choline, or at the N‐terminus close to the source of positive charge, representing the liquid‐state manifestation of the α‐ and ß‐ crystalline forms of choline chloride, respectively.[^39^, ^40^] Then, at the outside of the ‘cage’, malic acid molecules compete to form H‐bonds with chloride, each other, and choline, as is known for ChCl‐acid DES.^[41]^ As has been shown previously, all the ‘DES‐DES’ interspecies interactions are reduced slightly on addition of 2 mole equivalents of water, but the reduction is more in line with the water volume fraction than the mole fraction.^[14]^

**Table 1:** Coordination numbers for molecules and comparison with past literature data from Hammond et al.^*[14]^ Note that previous reports of ChCl:MA structure modelled the pure DES, whereas the systems studied here used a trace water content for the simulations (0.1 wt %), facilitating the comparison of DES‐water interactions at low concentrations.

A	B	*R*_max_ [Å]	ChCl : MA*	ChCl : Ma (ind)	ChCl : MA 2w*	ChCl : MA 2*w* (ind)
Choline	Malic Acid	8.5	6.78±1.93	6.76±1.97	6.22±1.78	5.65±1.91
Choline	Chloride	4.5	0.98±0.78	1.18±0.79	0.85±0.72	0.94±0.75
Choline	Choline	8.5	6.44±1.80	6.60±1.73	5.75±1.63	5.88±1.73
Malic Acid	Chloride	4.6	1.11±0.82	1.04±0.71	1.04±0.78	0.84±0.68
Malic Acid	Malic Acid	7.0	3.64±1.52	3.58±1.49	3.19±1.40	3.11±1.50
Chloride	Chloride	5.3	0.78±0.76	0.53±0.64	0.71±0.74	0.52±0.63
Choline	Water	4.1	–	0.01±0.10	0.94±0.90	0.91±0.88
Malic Acid	Water	4.3	–	0.01±0.10	1.47±1.20	1.16±1.02
Chloride	Water	4.2	–	0.02±0.14	2.05±1.26	2.05±1.35
Water	Water	3.8	–	0.00±0.01	1.54±1.24	1.44±1.14

Since the diffraction data when indole is present closely match previous studies of malicine DES,^[14]^ it is not surprising that there are mostly only minor deviations to note in terms of the bulk structure, on the addition of the hydrophobic solute. These are described in detail by the RDF integrals (coordination numbers; *N*_coord_) shown in Table 1, and their comparison to previous reports.^[14]^ Partial radial distribution functions for the bulk interactions were also calculated for comparison with the previous data, and are shown in the Supporting Information; the bulk pRDFs did not differ greatly from the bulk structure in the absence of solute.^[14]^ Similarly, the corresponding full set of concentration‐normalized RDF integrals (*N*_coord_) were calculated, showing only subtle differences in specific interactions, and these are also given in the Supporting Information. All changes to the solvation of solvent species on addition of the indole are soundly within the standard deviation margins. However, some intermolecular interactions do change slightly more when the hydrophobe is introduced, namely, Ch^+^−Cl^−^, and Cl^−^−Cl^−^. On addition of indole to malicine‐0*w*, the chloride‐chloride *N*_coord_ reduces from 0.78±0.76 to 0.53±0.64, and similarly for malicine‐2*w*, where *N*_coord_ reduces from 0.71±0.74 to 0.52±0.63. Meanwhile, the choline‐chloride *N*_coord_ increases from 6.44±1.80 to 6.60±1.73 for the 0*w* DES, and 5.75±1.63 to 5.88±1.73 for the 2*w* DES. Thus, introducing the hydrophobic indole solute induces a slight rearrangement of the solvation within the solvent itself, specifically the chloride‐based interactions; indole causes chloride to associate slightly more strongly with choline, both at the −OH group representing the choline chloride ‘α‐like’ interaction where Cl^−^ is H‐bonded with choline,^[39]^ and at the N atom, representing the choline chloride ‘ß‐like’ structural state.^[40]^ The water‐based interactions are negligible due to the trace concentration in the 0*w* DES, but interestingly the introduction of indole also causes malic acid to associate noticeably less strongly with water, as the malic acid‐water *N*_coord_ reduces from 1.47±1.20 to 1.16±1.02. Overall, the solvation of the hydrophobe does induce some subtle rearrangements in the bulk structure, which seem difficult to predict due to the many components present in the mixtures. Similar effects were observed for the solvation of transition‐ and rare earth‐metal ions in DES.[^42^, ^43^] Further investigations of the precise mechanisms of indole solvation are therefore warranted.

Another interesting detail, shown in the inset to Figure 3, arises from the calculated Kirkwood‐Buff integrals for the H_2_O−Cl interaction. This function is the spatial integral of the pair distribution function, allowing the quantification of the excess of species *j* around species *i*.^[44]^ Within the RDF cutoff range, we observe a slight enrichment of chloride around water molecules, with KBI of 279 and 56 Å^3^ (per molecule) for the 0*w* and 2w systems, respectively. In other words, this provides evidence for fluctuating nanoscale heterogeneities within the DES, due to close association of water and chloride, especially for the ‘dry’ DES system, at around 7.5 Å. This is significant because it aligns with MD and NMR observations by Harries et al.,^[45]^ and Di Pietro et al.,^[35]^ who showed ‘pearl‐on‐string’ solvation clusters, where chloride is preferentially solvated by water, and forms bridges to nearby water molecules through the solvation sphere.^[46]^ The KBI is less pronounced for the hydrated system, because the water content is higher and thus more evenly‐distributed through the bulk.

### Solvation of indole

**Figure 5:**
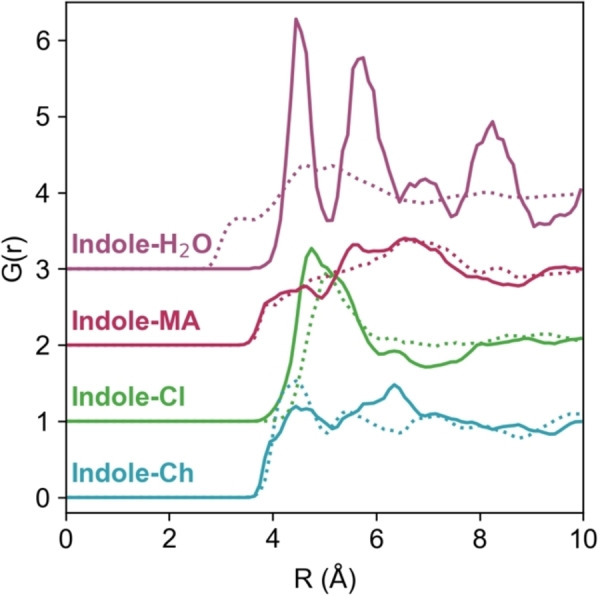
COM RDFs for the various molecular species around indole, for the 0*w* system (solid lines) and 2*w* solvent (dotted lines). As above, the high degree of fluctuations for the Indole‐H_2_O RDF in the low‐water system reflects the very low concentration of water used for the simulation, and they are thus occasionally found in preferred environments in the intermediate distance range away from the indole centre. As seen in for example the N_i_−O_1_ *N*_coord_ in Table 2, the actual number of involved water molecules is very small.

Having established that the hydrophobe has a minor effect on the various bulk interactions, we now turn our focus to the solvation of indole itself. The centre‐of‐mass RDFs for choline, chloride, malic acid and water around indole are shown in Figure 5, for the 0*w* and 2*w* DES. Indole is capable of interacting with all of the DES species, but in the COM data there are no sharp peaks with close‐range onsets. The most significant indole‐‘DES’ peak is the indole‐chloride interaction, which shows the possibility that indole can act as a secondary H‐bond donor. As before, due to the low concentration of both indole and water molecules, the indole‐water RDF for the 0*w* system has poor averaging statistics; running the simulation for much longer returns a flat line of zero for this result. However, the indole‐H_2_O RDF for the 2*w* DES has the closest onset of any of the COM‐RDFs, providing an initial suggestion that our hydrophobe, indole, becomes strongly hydrated as the water content of the DES increases to ca. 11.6 wt.%.

**Figure 6:**
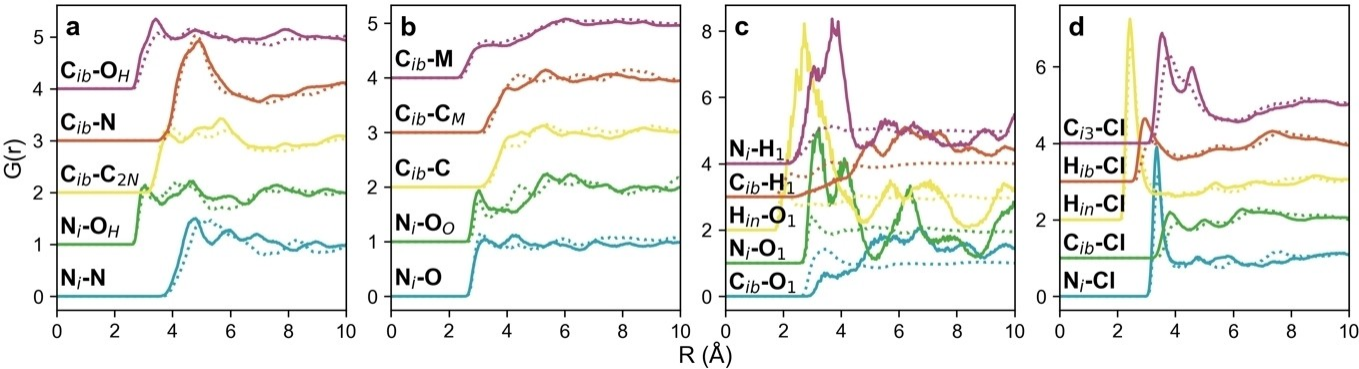
Partial (site‐specific) RDFs for a) choline, b) malic acid, c) water and d) chloride around indole, for the 0*w* system (solid lines) and 2*w* solvent (dotted lines).

To better understand the nature of the interactions, partial (site‐site) RDFs were calculated between molecular points of interest, and are shown in Figure 6. Some pRDF coordination numbers (*N*_coord_) were also calculated, signifying the number of atoms *j* around atom *i* within the first coordination shell, which are given in Table 2.

**Table 2:** Coordination numbers of the various sites calculated around indole atoms.

A	B	*R*_max_ [Å]	*N*_coord_ ChCl : MA	*N*_coord_ ChCl : MA : 2*w*
N_i_	O	3.6	0.61±0.71	0.53±0.71
N_i_	O_O_	3.6	0.23±0.45	0.14±0.35
C_ib_	C_M_	4.2	0.04±0.22	0.02±0.15
C_ib_	C	5.0	0.59±0.71	0.42±0.59
C_ib_	Cl	4.5	0.51±0.61	0.34±0.50
C_ib_	O_H_	4.1	0.56±0.70	0.40±0.55
C_ib_	C_2N_	4.0	0.51±0.69	0.49±0.59
N_i_	Cl	4.2	0.78±0.66	0.59±0.59
N_i_	O_H_	3.6	0.27±0.45	0.22±0.50
N_i_	O_1_	3.6	0.01±0.11	0.55±0.67

**Figure 7:**
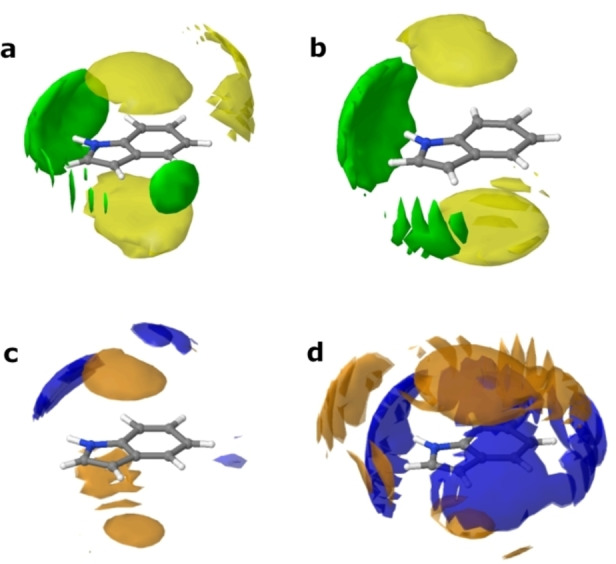
SDFs for the 0*w* DES (a, c) and the 2*w* DES (b, d), showing solvation of the central indole molecule, by cholinium ions (yellow), chloride (green), malic acid (orange) and water (blue) surfaces. The plotted isosurface fraction corresponds to the top 7.5 % of molecular configurations.

Firstly, we look at the hydration of indole, since this is key to understanding the details of how the other interactions change. A large, nonlinear increase in the coordination number for water around indole is observed, from 0.01±0.11 in the ‘dry’ 0*w* system with 0.1 wt.% H_2_O, to 0.55±0.67 in the ‘hydrated’ 2*w* system with 11.6 wt.% H_2_O. This is above the natural system stoichiometry, as water constitutes just 39.8 % of the box numerically, and much less in volume fraction. This is interesting, because indole itself is only sparingly soluble in water (ca. 1.9 g L^−1^ at 20 °C, or 1 : 3400 indole:water^[47]^), and while solubility data is not available for indole in these DES, no solubility issues nor phase separations were ever reported for indole in organic reaction studies in DES.^[27]^ Analysis of the SDFs in Figure 7(c and d) clarify that this increase is not just due to the ability of indole to act as an H‐bond donor for water: additionally, there is clear evidence of hydrophobic hydration of indole for the *2w‐*hydrated system. In the 0*w* system, water is only found out‐of‐plane with indole, with a main (albeit small) hydration lobe near the −NH group, signifying a ‘hydrophilic hydration’ regime.^[48]^ This is interesting when compared with previous studies, using neutron diffraction, of indole in amphiphilic water‐methanol mixtures by Johnston et al. and Henao et al., who found MeOH donates H‐bonds to the indole π‐system, and a lack of indole‐methyl directional interactions.[^47^, ^49^] However, here, for the 2*w* DES, we see that water closely surrounds the heterocyclic ring, encapsulating all of the hydrophobic aromatic protons, as well as having strong H‐bonding with the −NH group, and water apparently does not H‐bond with the π‐system. Consulting the SDFs in tandem with the indole‐water pRDFs in Figure 6(c), hydrophobic hydration of the indole molecule by the DES therefore perhaps counterintuitively increases at the pyrrole terminus as water content increases, and the solvation regime is clearly different from when there is only trace water, and different from the H_2_O/MeOH solutions. This is shown by the N_i_−H_1_ and N_i_−O_1_ pRDFs, since the first‐neighbour distance is closely related for both the H‐ and O‐ interactions, indicating the distinct tangential orientation of water molecules associated with the surface of the hydrophobe.^[50]^ Meanwhile, at the benzene terminus of indole, as water content increases, the C_ib_−O_1_ and C_ib_−H_1_ onset distances become more divergent, signifying more directionality. This may offer explanations for differing heterocyclic reaction pathways and outcomes in systems where the water content is completely uncontrolled. However, we can only define that this regime change occurs somewhere below 2 mole equivalents of water for this DES system. It has been observed that Grignard reactions are possible in some DES under ambient conditions;^[51]^ these results suggest that small quantities of absorbed atmospheric water, below a certain (low) threshold, are largely sequestered by the DES and do not interact significantly with the reactive solutes of interest.

Next, the distribution of chloride around indole was considered. In the opposite manner to the indole‐water correlations, the indole‐chloride *N*_coords_ are high for the 0*w* solvent, and reduced on addition of water. Namely, the N_i_−Cl and H_in_−Cl pRDFs, signifying indole donating an H‐bond to chloride, have intense close‐range peaks, and the N_i_−Cl *N*_coord_ is 0.78±0.66 in the 0*w* system. This value sees a significant reduction to 0.59±0.59 in the 2*w* DES. Consulting the SDFs in Figure 7(a and b), chloride has a well‐defined interaction with indole at the H‐bonding −NH group, and a secondary one at an oppositely‐situated, slightly acidic indole aromatic proton site, and these distributions both become much more diffuse on addition of water. Thus, indole functions as a secondary HBD molecule in the DES, which is why it induces rearrangements in the bulk structure, but this ability is diminished on addition of water, since water competes for the same role, relegating indole to back‐seating as a ‘tertiary HBD’.

The indole‐choline interactions show no enormous difference in any of the *N*_coord_ values, but it is clear that the addition of water causes a substantial rearrangement of choline around indole. Namely, The C_ib_−OH (indole aromatic **C**−choline −**O**H group) interaction decreases from 0.56±0.70 to 0.40±0.55, while the indole N_i_ group interacts minimally with the choline −**O**H at 0*w*, and reduces even further in the 2*w* solvent, in line with water volume fraction. We can conclude from this information that there is not a significant amount of H‐bonding between indole and choline; i. e., for the 0*w* system with a mean coordination number of 0.56, 44 % of indole molecules are not bound to choline, and 56 % are bound to one cation. However, at the same time, the C_ib_−C_2N_ coordination number is apparently conserved as water content increases, varying only from 0.51±0.69 to 0.49±0.59, with even a slight reduction in the standard deviation. The SDFs shown in Figure 7(a, b) clarify that this is due to the disappearance of the perpendicular solvation lobe of choline around indole which exists in the 0*w* system; in the 2*w* DES, choline is focused exclusively above and below (parallel, and in‐plane with) the indole aromatic rings. This arrangement is more electrostatically‐favoured, due to interactions between the N^+^ group and the π‐system.

Finally, we consider the interactions between malic acid and indole. Universally, malic acid does not interact strongly with indole in the 0*w* system, and this interaction is reduced even further on dilution of the DES with water. The most significant *N*_coord_ values are seen for N_i_−O (indole **N**−malic acid −CO**O**H), which are 0.61±0.71 and 0.53±0.71 for the 0*w* and 2*w* systems, respectively, and C_ib_‐C (indole aromatic **C**−malic acid backbone **C**), where *N*_coord_ is respectively 0.59±0.71 and 0.42±0.59 for the 0*w* and 2*w* systems. There is thus minimal interaction between indole and the malic acid −**O**H group, but some slight H‐bonding is possible with the carboxylic acid O. When the system is ‘dry’, malic acid sits perpendicular to the indole aromatic planes (Figure 7c), forming a sandwich structure with choline, which occupies the same space around the π‐system due to electrostatic interactions, and more proximal species (Cl^−^ or H_2_O), which are more closely associated with indole through H‐bonding. Increasing the water content has the interesting effect of making the malic acid distribution broader in the aromatic planes (Figure 7d), but also adding an additional lobe in‐line with the indole −NH group, at a greater distance.

## Conclusion

The solvation of indole, a prototypical organic synthesis and biomolecular proxy heterocycle, was investigated in the choline chloride–malic acid DES, with variable water content. The goal was to evaluate the bulk situation of the molecule. It has been observed that water can induce the sequestration of hydrophobic molecules into hydrophobic regions of a DES with significant hydrophobic character, but it is not clear if the same process can occur for hydrophilic DES.

The most important interactions governing the bulk structure of the DES are largely unchanged by the addition of indole. There is still significant H‐bonding between all available H‐bond donor sites and chloride, and it is irrelevant whether these are situated on the cation, or HBD. Chloride can be found either H‐bonded with the −OH group of choline, representing choline chloride's crystalline α‐form, or in close proximity with N^+^, i. e., the source of positive electrostatics, representing the ß‐crystalline form of choline chloride. These multiple accessible structural states likely contribute to the low eutectic points seen for ChCl‐based DES, through entropic contributions, making the potential energy landscape of interactions broad. Addition of water, when considered in terms of mole fraction, generally causes below‐expected reductions in the interactions between DES components.

The solvation of indole itself in this DES was found to be subject to interesting composition‐dependent effects, and especially in comparison to previous work, using the same diffraction technique, of indole solvation in water/methanol solutions.[^47^, ^49^] Here, we do not observe any water H‐bond donation to the indole aromatic π‐system. Rather, choline occupies the spaces above and below the aromatic plane, driven by electrostatic interactions, and is associated with malic acid in a ‘sandwich’ structure. Indole is capable of functioning as a secondary H‐bond donor, with its accessible −NH proton donating to the chloride anion, but the addition of water relegates indole to a ‘tertiary’ HBD, and it is outcompeted by the strong association between water and chloride. Most interestingly, as water content is increased, water solvates the indole strongly as the solvation regime changes; weak H‐bonds are observed in the plane of the heterocycle, in addition to the strong H‐bonding between water and indole at the −NH site.

Overall, the results presented here provide interesting information on the solvation of small‐molecule hydrophobes in general, whether that is as directly in an organic reaction involving indole, or as a proxy for a related heterocyclic molecule. These data are also of interest for those studying the solvation of cellular metabolites, and proteins containing the tryptophan residue, in complicated mixed solvation media such as DES.

## Experimental Details

Detailed descriptions of sample preparation, neutron diffraction and atomistic modelling techniques are provided in the Supporting Information.

## Conflict of interest

The authors declare no conflict of interest.

1

## Supporting information

As a service to our authors and readers, this journal provides supporting information supplied by the authors. Such materials are peer reviewed and may be re‐organized for online delivery, but are not copy‐edited or typeset. Technical support issues arising from supporting information (other than missing files) should be addressed to the authors.

Supporting Information

## Data Availability

Raw experimental neutron diffraction data are available through the ISIS‐ICAT system, DOI: 10.5286/ISIS.E.RB1720287. Reduced, treated diffraction datasets, to which the atomistic EPSR models were refined, are available through the University of Bath research data archive system, DOI: https://doi.org/10.15125/BATH‐01138
