# Supplementary material for: Neutron Diffraction Study of Indole Solvation in Deep Eutectic Systems of Choline Chloride, Malic Acid, and Water
Source: Chemistry. 2022 Jun 13;28(41):e202200566. doi: 10.1002/chem.202200566 (PMC9400976; doi:10.1002/chem.202200566)
Supplement: Supplementary file 1 — Supporting Information [file CHEM-28-0-s001.pdf]

Chemistry–A European Journal

Supporting Information

Neutron Diffraction Study of Indole Solvation in Deep Eutectic Systems of Choline Chloride, Malic Acid, and Water

Oliver S. Hammond,* Ria Atri, Daniel T. Bowron, and Karen J. Edler*

Experimental Details

Preparation of samples

Choline chloride (≥98%) and malic acid (≥99%) were purchased from Sigma-Aldrich and dried under vacuum prior to use. Deuterated equivalents, namely trimethyl-d_9-choline chloride ($(CD_3)_3N(CH_2)_2OHCl$); 98 atom % D) and 2,3,3-d_3-(*DL*)-Malic acid ($(HO_2CCD_2CD(OH)CO_2H$; 98 atom % D) were also purchased from Sigma-Aldrich and used without further purification. Anhydrous eutectic mixtures were prepared by mixing the measured components together in a 1:1 molar ratio, with agitation, and heating for the minimum possible time at 80°C. A sample of ChCl:malic acid was prepared without any special control over water content, and measured using a Bohlin CVO 120 rheometer with 40 mm plate with 4° angle and 150 µm gap, across a shear stress range of 1 – 50 Pa. This sample had a viscosity of 18277 mPa.s, making ChCl:malic acid one of the most viscous DES reported to date, and potentially even more viscous when prepared under completely dry conditions. Thus, despite the high concentrations of potential reactants, we are not concerned about the system degrading markedly during preparation or measurement, due to its incredibly high viscosity inhibiting diffusion rates, and thus the esterification reaction kinetics. Hydrated eutectic mixtures were then made either by adding the required amount of water to the prepared liquid 'dry DES' with mixing and without heating, or through the same mixing procedure starting with solids and water, at lower temperature (60°C) owing to the faster mixing (5-10 minutes total) afforded by the added water. The strong agreement between the neutron diffraction data and fits indicates that this preparation approach is robust, and despite any potential reactivity, our DES systems were of high purity and not reactive under these conditions. For the dry system, five (ChCl:malic acid) isotopic contrasts were therefore prepared, H:H, D:H, D:D, H:D, and H/D:H. For the hydrated system, nine (ChCl:malic acid:water) contrasts were prepared, of H:H:H, D:H:D, D:D:D, H:D:D, H/D:H:D, H:H:D, H/D:H:H/D, H:H:H/D, and D:H:H/D. 'H/D' indicates a 1:1 isotopic molar ratio. Protonated indole (Acros Organics, 99+%) was then added to each mixture to achieve a DES:indole concentration of approximately 50:1. Owing to the costly nature of the deuterated samples the "dry" samples were immediately reused to prepare the hydrated samples, so water contents were not systematically measured, yet it is known that such samples are hygroscopic.[1] Therefore, it was assumed that the dry systems contained a small quantity of water (*ca*. 0.1 wt.%) H_2O, as a reasonable estimate of water content, to improve the physical representation given by the modelling.

Neutron diffraction

The NIMROD instrument was used to make wide Q-range neutron diffraction measurements of the indole-containing DES solutions.[2] Its significant Q-range of $0.01 \leq Q \leq 50$ Å^{-1} is facilitated by a broad detector coverage of $0.6° \leq \theta \leq 37.5°$, and time-of-flight neutrons spanning wavelengths of $0.05 \leq \lambda \leq 11$ Å, meaning that the instrument can measure real-space length scales spanning over two orders of magnitude, from 0.1 – 300 Å.

Each sample was placed into vacuum-sealed 1 mm thick flat-plate $Ti_{0.68}Zr_{0.32}$ alloy cells and regulated to 30 ± 0.1 °C in the instrument sample changer. Measurements were made of the sample-containing cells, alongside the empty instrument, empty cells, and a 3 mm vanadium standard, for a median of 2 h. GudrunN was then used to reduce the raw data.[3] The data processing workflow involves correcting for the background, attenuation, and multiple scattering, then normalizing to absolute units before subtracting the residual hydrogen inelastic scattering signal iteratively. These datasets were then used for refinement using Empirical Potential Structure Refinement (EPSR).

Atomistic Modelling

The Empirical Potential Structure Refinement package (EPSR25) was used for analysis of the data. This procedure is well-described elsewhere,[4–6] and is an established method for the determination of structure in disordered phases, including complex liquids such as DES.[7,8] Lennard-Jones parameters and atom names for hydrated malic acid DES are exactly as reported previously,[8] and indole parameters and labels are the same as described by McLain *et al.*, who studied its solvation in water-methanol solutions.[9]

Simulation boxes containing *ca*. 100,000 atoms were constructed in representative molar ratios; the box dimensions and compositions are detailed in Table S1 below. Since the water content could not be routinely controlled experimentally due to the presence of scarce deuterated components, 0.1 wt.% H_2O was also added in the 'dry' model to better represent the physicality of the system. Such large simulation boxes were favoured due to the low concentration of the solute of interest, to accumulate sufficiently good counting statistics for radial distribution functions, coordination numbers and spatial density functions. Ensemble average information was collected for approximately 10,000 box configurations, following system equilibration, under the reference + empirical potential framework.

The discussion section shows and compares calculated "errors" in the coordination number (N_{coord}). However, these are not simple errors that can be interpreted as an atom having no

neighbours of the specified type within the quoted uncertainty (*c.f.* 0.53 ± 0.64). Rather, this reflects the range of environments that a particular atomic centre finds itself in. The quoted N_{coord} is therefore the mean N_{coord}, determined from the many thousands of sites and simulation box iterations that have been interrogated during the ensemble averaging process. The "error" therefore is more representative of the variability of the local environment, and is thus the uncertainty in the number of neighbours.

Table S1. Compositions of the EPSR simulation boxes used in this study.

**Quantity**	**'Dry' (0*w*)**	**'Hydrated' (2*w*)**
Choline	2750	2363
Chloride	2750	2363
Malic acid	2750	2363
Indole	55	47
Water	42	4726
Molecules	8347	11862
Atoms	99256	99340
Box diameter (Å)	100.91037	100.78090
Box volume (10^{-6} Å^3)	1.02756	1.02361
Accumulated EPSR iterations	9765	9513

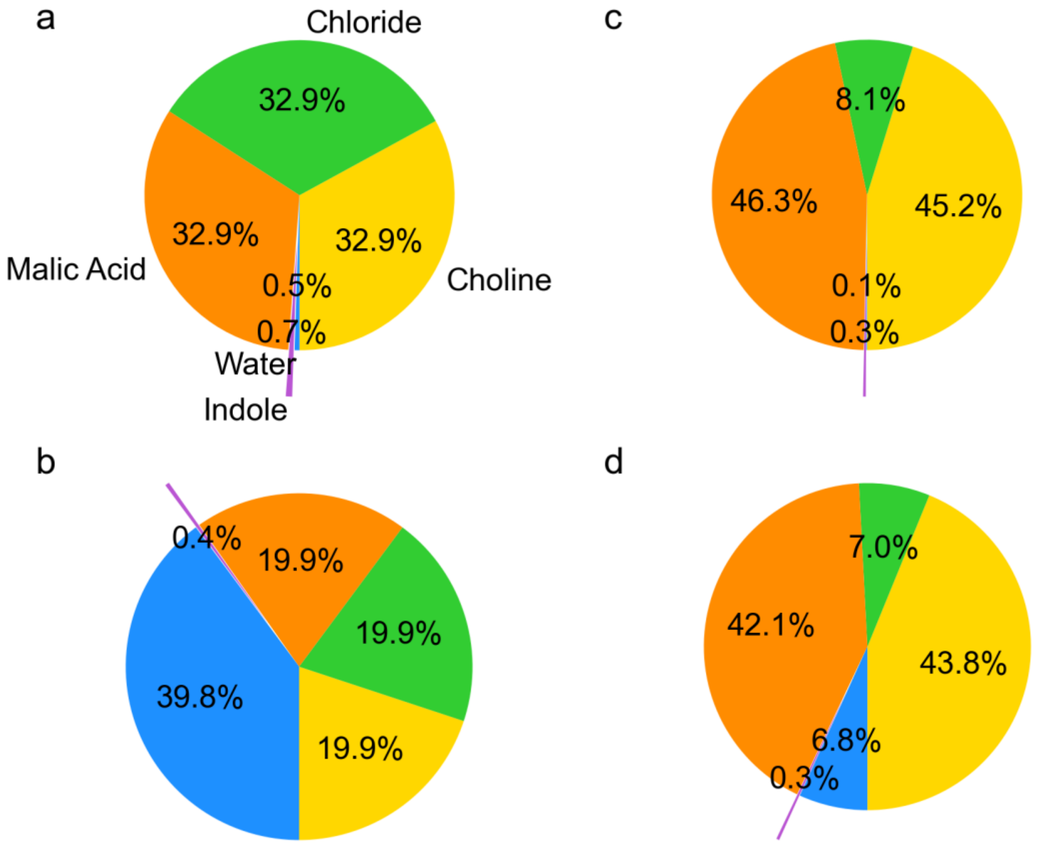

Figure S2. Comparison of the fraction of each species in the first solvation shell around a central choline molecule, for the 'ideal' distributions (a) and (b), which correspond with the random distributions in a local volume, and are taken from the numerical composition of the simulation box for the 0*w* and 2*w* systems respectively; and the actual calculated fractional coordination coordination from the EPSR simulations (c) and (d) for 0*w* and 2*w* DES respectively. The yellow portion represents choline, orange is malic acid, green is chloride, blue is water, and the exploded purple portion is indole.

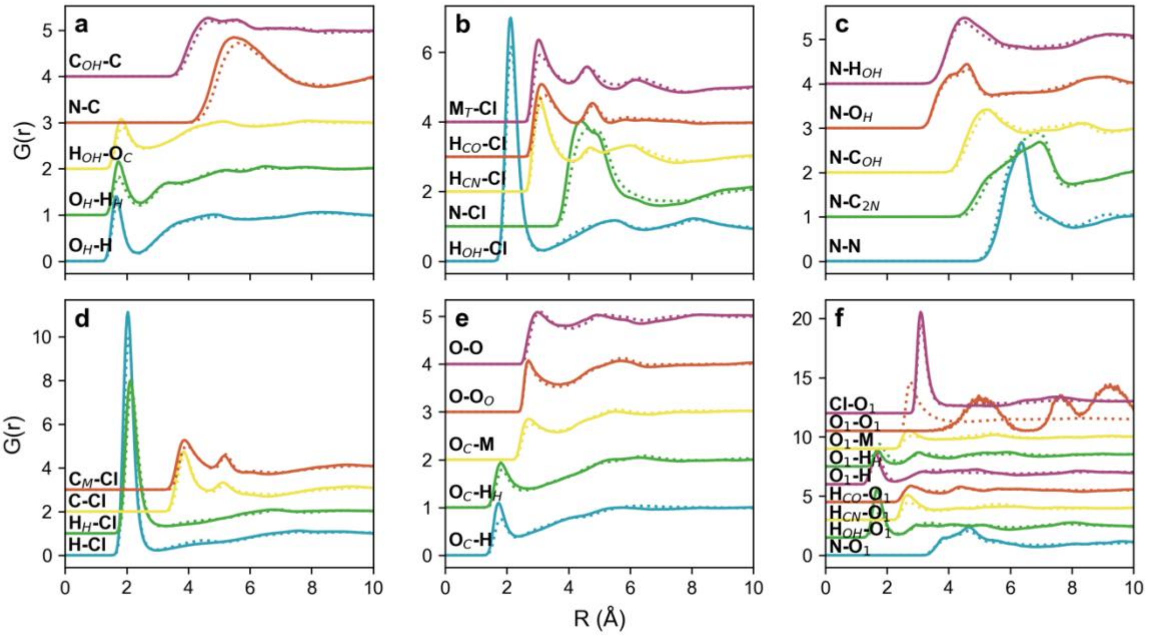

Figure S3. Partial radial distribution functions for the bulk solvents, representing specific interactions for (a) choline-malic acid, (b) choline-chloride, (c) choline-choline, (d) malic acid-chloride, (e) malic acid-malic acid, (f) various water-based interactions. Solid lines are for the pure (unhydrated) system, whereas dotted lines show the 2*w* water composition.

Table S4. Calculated partial (site-site) coordination numbers, and comparison with past literature data.[8]

	A	B	R_{max} (Å)	ChCl:MA(lit*)	ChCl:Ma(ind)	ChCl:MA2w(lit*)	ChCl:MA2w(ind)
Cho-Mal	O_H	H	8.5	0.13±0.34	0.13±0.34	0.14±0.35	0.11±0.31
	O_H	H_H	4.5	0.07±0.25	0.07±0.25	0.03±0.17	0.04±0.21
	H_{OH}	O_C	8.5	0.16±0.37	0.15±0.36	0.11±0.32	0.11±0.32
	N	C	4.6	5.85±1.32	5.85±1.82	5.21±1.66	4.76±1.73
	C_{OH}	C	7.0	2.37±1.16	2.33±1.23	2.13±1.15	1.90±1.16
Cho-Cl	H_{OH}	Cl	5.3	0.52±0.51	0.58±0.50	0.48±0.51	0.45±0.50
	N	Cl	4.1	3.22±1.19	3.41±1.14	2.86±1.15	2.99±1.11
	H_{CN}	Cl	4.3	0.65±0.64	0.77±0.65	0.56±0.61	0.64±0.62
	H_{CO}	Cl	4.2	0.66±0.66	0.72±0.65	0.58±0.65	0.55±0.61
	M_T	Cl	3.8	0.59±0.62	0.63±0.62	0.48±0.58	0.49±0.57
Cho-Cho	N	N	8.0	5.35±1.63	5.27±1.51	4.59±1.47	4.63±1.55
	N	C_{2N}	8.0	6.39±1.67	6.40±1.60	5.72±1.63	5.80±1.56
	N	C_{OH}	6.0	2.84±1.20	2.75±1.18	2.66±1.08	2.45±1.09
	C	O_H	5.2	2.19±1.00	1.20±0.99	2.01±0.99	0.98±0.91
	N	H_{OH}	6.0	3.01±1.28	3.07±1.32	2.75±1.12	2.65±1.15
Mal-Cl	H	Cl	3.1	0.62±0.51	0.69±0.47	0.54±0.53	0.56±0.50
	H_H	Cl	3.3	0.56±0.54	0.61±0.50	0.53±0.50	0.52±0.50
	C	Cl	4.6	1.11±0.82	1.04±0.71	1.03±0.78	0.84±0.68
	C_M	Cl	4.6	1.06±0.80	0.99±0.76	0.98±0.81	0.76±0.69
Mal-Mal	O_C	H	2.3	0.25±0.47	0.21±0.43	0.22±0.43	0.14±0.36
	O_C	H_H	2.6	0.17±0.39	0.13±0.35	0.15±0.38	0.09±0.30
	O_C	M	3.3	2.59±1.01	2.92±0.97	2.70±0.91	2.79±0.91
	O	O_O	3.7	0.93±0.70	0.83±0.72	0.86±0.65	0.77±0.67
	O	O	3.7	0.73±0.78	0.67±0.75	0.63±0.75	0.56±0.70
Cl-Cl	Cl	Cl	5.3	0.78±0.76	0.53±0.64	0.71±0.74	0.52±0.63
Cho-H_2O	N	O_1	6.0	-	0.04±0.21	4.94±2.10	4.64±2.10
	H_{OH}	O_1	2.3	-	0.00±0.04	0.17±0.39	0.19±0.39
	H_{CN}	O_1	3.6	-	0.01±0.09	0.65±0.78	0.64±0.75
	H_{CO}	O_1	3.6	-	0.01±0.07	0.73±0.78	0.65±0.77
Mal-H_2O	H	O_1	2.3	-	0.00±0.04	0.19±0.39	0.22±0.41
	H_H	O_1	2.3	-	0.00±0.03	0.12±0.33	0.19±0.40
	M	O_1	3.6	-	0.01±0.08	0.73±0.79	0.55±0.72
Cl-H_2O	Cl	O_1	4.2	-	0.02±0.14	2.05±1.26	2.05±1.35
H_2O-H_2O	O_1	O_1	3.8	-	0.00±0.01	1.54±1.24	1.44±1.14

References

[1] X. Meng, K. Ballerat-Busserolles, P. Husson, J.-M. Andanson, *New J Chem* **2016**, *40*, 4492–4499.

[2] D. T. Bowron, A. K. Soper, K. Jones, S. Ansell, S. Birch, J. Norris, L. Perrott, D. Riedel, N. J. Rhodes, S. R. Wakefield, A. Botti, M.-A. Ricci, F. Grazzi, M. Zoppi, *Rev. Sci. Instrum.* **2010**, *81*, 033905.

[3] A. K. Soper, *GudrunN and GudrunX: Programs for Correcting Raw Neutron and X-Ray Diffraction Data to Differential Scattering Cross Section. Rutherford Appleton Laboratory Technical Report RAL-TR-2011-013*, **2011**.

[4] A. K. Soper, *Chem. Phys.* **1996**, *202*, 295–306.

[5] A. K. Soper, *Mol. Phys.* **2001**, *99*, 1503–1516.

[6] A. Soper, "Empirical Potential Structure Refinement (ISIS)," can be found under: http://www.isis.stfc.ac.uk/groups/disordered-materials/downloads/empirical-potential-structure-refinement6157.html, **2015**.

[7] O. S. Hammond, D. T. Bowron, K. J. Edler, *Green Chem* **2016**, *18*, 2736–2744.

[8] O. S. Hammond, D. T. Bowron, A. J. Jackson, T. Arnold, A. Sanchez-Fernandez, N. Tsapatsaris, V. G. Sakai, K. J. Edler, *J Phys Chem B* **2017**, *121*, 7473–7483.

[9] A. Henao, A. J. Johnston, E. Guàrdia, S. E. McLain, L. C. Pardo, *Phys. Chem. Chem. Phys.* **2016**, *18*, 23006–23016.
